# Factors Associated with Hypercementosis: A Cone Beam Tomography Study of Local Dental Conditions

**DOI:** 10.4317/jced.63643

**Published:** 2026-01-28

**Authors:** María Victoria Martiren, Jhoana Mercedes Llaguno-Rubio, Gustavo Adolf Fiori-Chincaro, Luis Ernesto Arriola-Guillén

**Affiliations:** 1 Division of Oral and Maxillofacial Radiology, Faculty of Dentistry, Centro Universitário do Norte de São Paulo (UNORTE), São Paulo, Brazil; 2 Associate Professor, Division of Oral Radiology, Instituto Latinoamericano de Altos Estudios en Estomatología (ILAE), Lima, Peru; 3 Associate Professor, Division of Oral Radiology, Instituto Latinoamericano de Altos Estudios en Estomatología (ILAE), Lima, Peru; 4 Ph.D. and Associate Professor, Division of Orthodontics, Faculty of Dentistry, Universidad Científica del Sur, Lima, Peru

## Abstract

**Background:**

To evaluate the morphological characteristics of hypercementosis and its association with local factors using cone beam computed tomography (CBCT) in a population from Uruguay.

**Material and Methods:**

This observational, cross-sectional, retrospective study analyzed CBCT scans from 1,830 patients aged 25 and older. The study recorded the presence and type of hypercementosis (diffuse, focal, maggot-shaped), the types of teeth affected, and associated local factors, including the absence of an antagonist, dental impaction, occlusal trauma, periodontal disease, periapical processes, and endodontic or orthodontic treatments, among others. Statistical analysis included chi-square tests and multiple linear regression, with a significance level set at p &lt; 0.05.

**Results:**

Hypercementosis was observed in 19% of the patients, affecting 595 teeth, primarily upper molars (48.6%) and lower molars (23.6%). The diffuse type was the most prevalent, accounting for 93.3% of cases. The absence of an antagonist tooth was identified as the most common local factor (24.71%), followed by dental impaction (21.01%) and idiopathic cases (13.28%). A significant association was found between the type of hypercementosis and the affected teeth (p &lt; 0.001), as well as between local factors and morphological presentation (p &lt; 0.001). Linear regression analysis indicated that male sex was a protective factor for fewer affected teeth (p = 0.010), whereas age had no significant effect (p = 0.273).

**Conclusions:**

The absence of an antagonist tooth was the primary local factor associated with hypercementosis, with the diffuse form predominating across all dental groups. Male sex appears to be a protective factor concerning the number of affected teeth, while age showed no significant association. These findings underscore the importance of considering functional and local factors in diagnosing and planning endodontic, orthodontic, and surgical treatments for patients with hypercementosis.

## Introduction

Hypercementosis is a non-neoplastic condition characterized by excessive deposition of root cement beyond normal levels (1). Macroscopic changes in the root of the tooth are evident (2 , 3). This condition is typically asymptomatic, and its diagnosis is based on radiographic findings (4). On radiographs, one may observe partial or total thickening of the root contour, while the lamina dura and periodontal ligament remain intact (5 , 6). Morphologically, hypercementosis can be diffused, focal, or sleeve-shaped, with the diffuse form being the most commonly observed (1 - 3 , 7). Its etiology is considered idiopathic and may be related to the patient's age (1 , 8); however, it is also associated with local factors such as the absence of an antagonist tooth, occlusal trauma, chronic periodontal inflammation, tooth impaction, and various systemic factors (3 , 6 , 8 - 10). Literature indicates that hypercementosis is a common condition, although it is rarely reported (1 , 2 , 7). Recent studies reveal prevalence rates of 16.3% in the Mexican population, 10.8% in the Turkish population, 9.8% in the Saudi Arabian population, 2.4% in southeastern Turkey, 1.3% in the German population, and 0.2% in the Iranian population (5 , 11 - 15). It is found to occur more frequently in mandibular molars and premolars (1 , 5 , 11 , 12 , 15). Hypercementosis usually does not require treatment; however, it is important to recognize this condition because it can lead to complications during endodontic (root canal) and orthodontic procedures, as well as during tooth extractions. Understanding hypercementosis is particularly crucial since the apical foramen can be in different positions (2). Additionally, there is a risk of partial ankylosis (the fusion of the tooth to the surrounding bone) associated with hypercementosis (8). Therefore, this study aims to evaluate the characteristics and types of hypercementosis in relation to local associated factors using cone-beam computed tomography (CBCT). This approach could improve the assessment and classification of the condition.

## Material and Methods

**Figure 1:**
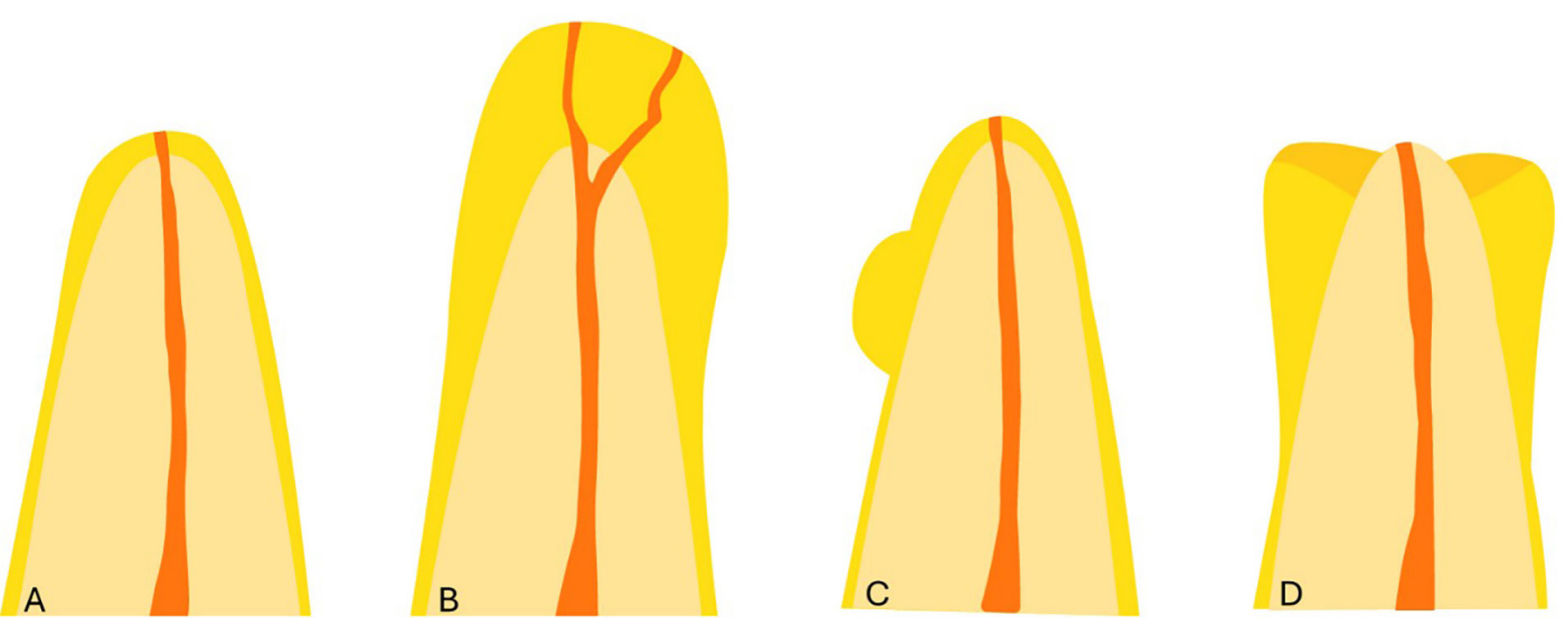
Morphological types of hypercementosis: A) Normal root; B) Diffuse hypercementosis; C) Focal or localized hypercementosis; D) Sleeve-shaped hypercementosis.

**Figure 2:**
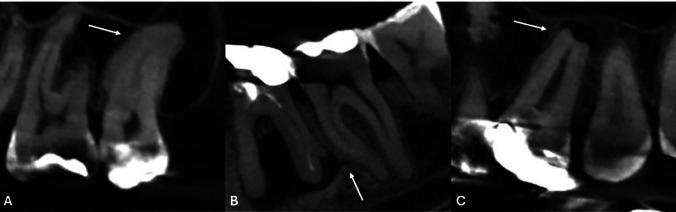
The arrows indicate: A) Diffuse hypercementosis in the buccal roots of an upper molar. B) Focal hypercementosis located in the middle third of the mesial root of a lower molar. C) Sleeve-like hypercementosis observed in the palatal root of an upper molar.

- Study design This study obtained authorization and ethical approval from Centro Universitário do Norte de São Paulo (UNORTE), São Paulo, Brazil. We utilized a cross-sectional, observational, and retrospective design. The study group included 2107 CBCTs images. - Sample size calculation The sample size was calculated using a formula to estimate the proportion of patients with hypercementosis in the absence of an antagonist tooth, set at 25% based on data from a previous pilot study. A precision level of 3% indicated a minimum required sample size of 800 patients; however, the final study included 1830 patients. - Selection criteria We included CBCT scans from adult patients, aged 25 years and older, of both genders, who were treated at a private radiological imaging center in Montevideo, Uruguay, between 2018 and 2024. The scans could clearly show identifiable teeth with hypercementosis. We excluded any scans that did not meet quality standards, such as those affected by movement, noise, flashes, or artifacts. Additionally, scans with orthodontic or rehabilitation appliances, or those demonstrating pathologies or surgeries that could hinder accurate observation, were also excluded. - Image Acquisition All images were obtained using a Trophy brand CBCT device, model Trophypan Smart SC 3D, CS 8100, manufactured in Finland in 2017. The fields of view measured 5 cm x 5 cm, 5 cm x 8 cm, and 8 cm x 9 cm, with a voxel size resolution of 150 m. The device was set to a Kv of 60-90, an mA of 2-15, and an exposure time of 7-15 seconds. The patient's position was adjusted per the manufacturer's manual, ensuring the Frankfort plane was parallel to the floor and that the bite and positioning support points were correct. - Reading and Recording Images were evaluated in a controlled, semi-lit environment using a 32-inch Samsung M8 flat-screen monitor. Evaluations were conducted between 7:00 a.m. and 10:00 a.m. All data was recorded in an Excel database according to the specified criteria. - Training and Pilot Test Prior to conducting the study, the examiner underwent a training phase using a randomly selected set of CT scans, focusing on radiographic identification of hypercementosis and on standardizing the CBCT evaluation procedure, under the guidance of an oral and maxillofacial radiology specialist with more than 5 years of experience. An intra- and inter-observer agreement index (kappa coefficient) of 0.9 or higher was achieved. Subsequently, a pilot test involving 10% of the study sample was conducted, allowing for adjustments in the observation order and confirming the feasibility of the proposed methodology. Results from the pilot test were included in the final analysis. - Variables measurements Hypercementosis was evaluated based on three types: diffuse (characterized by a club-shaped root), focal or localized (appearing as an isolated root surface), or sleeve-shaped (where the apex is laterally surrounded but not compromised at its most apical part), as illustrated in Figure 1 and 2.

1

2

We considered the subjects' age (in years) and sex (female or male). Additionally, we evaluated local triggering factors, which included: Dental impaction (when teeth have not erupted due to insufficient space, malposition, or obstructive elements). Absence of an antagonist tooth (either partial or complete retention of the opposing tooth). Occlusal trauma (resulting in faceting of the tooth surface). Periodontal disease (characterized by loss of alveolar bone height and increased periodontal ligament space). Periapical inflammation (identified by osteolytic images associated with the root of the tooth). Increased surrounding bone density, indicating signs of a periapical lesion. Existence of endodontic or orthodontic treatment. - Statistical Analysis Statistical analyses were conducted using SPSS version 19 for Windows. We examined the association between the number of hypercementosis cases and various factors, including sex, age, and local dental conditions, utilizing the chi-squared (²) test. Additionally, we employed multiple linear regression to assess whether the incidence of hypercementosis was influenced by age and sex. All analyses were performed using a significance level of p &lt; 0.05.

## Results

A total of 1,830 patients were evaluated, with hypercementosis observed in 348 (19%) and affecting 595 teeth overall. Table 1 presents the number of teeth with hypercementosis by sex, with a p-value of 0.156, indicating no statistically significant association.

Table 1

Most patients had hypercementosis in a single tooth, accounting for 57.2% of the total (199 patients). Within this group, females predominated with 138 patients (62.7%), while males accounted for 61 patients (47.7%). Additionally, 27.3% of patients had two affected teeth (24.5% in females and 32% in males), while up to 7 teeth with hypercementosis were found in a single patient (0.3%). Regarding the types of teeth affected by hypercementosis, the most commonly affected were the upper molars (169 teeth, 48.6%), followed by lower molars (82 teeth, 23.6%) and upper premolars (57 teeth, 16.4%). Upper canines were more affected than lower premolars (27 teeth, 7.8%, compared to 11 teeth, 3.2%). No significant association was found between sex and the type of tooth affected (p=0.272) (Table 2).

Table 2

Concerning the morphological patterns of hypercementosis, the diffuse form was the most prevalent, accounting for 91.7% (319 teeth), followed by focal hypercementosis at 6% (21 teeth) and the sleeve form at 2.3% (8 teeth). There was no significant association between sex and the type of hypercementosis (p=0.991) (Table 3).

Table 3

As shown in Table 4, there was a significant association between the type of hypercementosis, and the teeth evaluated (p &lt; 0.001). The diffuse form predominated across all tooth types, except the lower incisors, where focal hypercementosis was more common (66.7%).

Table 4

The highest number of focal cases was found in upper and lower molars (8 teeth in each group) and upper canines (4 teeth). Sleeve-shaped hypercementosis was more common in lower molars (6 teeth, 4.4%), followed by upper molars (2 teeth). When examining the association between hypercementosis type and contributing factors, the most frequently observed factors were: first, the absence of an antagonist (147 affected teeth); second, tooth impaction (125 teeth); and third, idiopathic cases (79 teeth). The type of hypercementosis was mainly diffuse, with 99.3%, 98.4%, and 97.5% prevalence for these factors, respectively. Other factors had less influence. The significance testing showed that combinations of specific factors-such as apical lesions, endodontic treatment, and increased bone density-can lead to focal or sleeve-type hypercementosis (Table 5).

Table 5

In exploring the relationship between the type of tooth with hypercementosis and related local factors, significant results were observed (p&lt;0.001). The most affected teeth in the absence of antagonist were primarily upper molars (81%). In cases of impaction, the upper molars were affected in 47.2% and the lower molars in 44.8%. For cases without local factors (idiopathic), the distribution among dental groups showed that upper premolars were most affected (34.2%), followed by lower molars (21.5%), upper canines (16.5%), upper molars (15.2%), and lower premolars (8.9%) (Table 6).

Table 6

Table 7 presents a multiple linear regression analysis evaluating the influence of sex and age on the number of teeth with hypercementosis. The analysis indicated that sex significantly influences this occurrence (p=0.010), demonstrating that being male is a protective factor against increased number of teeth with hypercementosis in 69.5% of cases.

Table 7

## Discussion

There is limited research examining the relationship between different types of hypercementosis and the local factors that contribute to them. Most studies are based on small retrospective studies of X-rays or on individual case reports. Recognizing the prevalence and associated factors of this condition is crucial because it can lead to complications during endodontic (root canal) treatments, orthodontic procedures, and tooth extractions. Understanding hypercementosis is essential, as the position of the apical foramen may vary, which can impact the treatment of affected teeth (16 - 20). Furthermore, there is a risk of partial ankylosis, the fusion of the tooth to the surrounding bone, associated with hypercementosis. In our study, the idiopathic factor was the third most prevalent (present in 13.3% of cases), unlike in Mexican (5) and Turkish (12) populations, where it was the most prevalent. The most frequent factor in our evaluation was the absence of an antagonist tooth (24.71%), in which occlusal forces are almost nil, and cement overproduction can be explained, according to Consolaro et al. (20), as an adaptive response to increase tooth-bone fixation. In this regard, a study conducted in Tokyo observed that functional teeth have a lower frequency of hypercementosis, suggesting that continuous occlusal forces prevent cement formation, thereby favoring the absorption of masticatory forces. The second factor evaluated in our study was tooth impaction (21,01%). As in the study conducted in the Mexican population, impaction ranked second in prevalence (23.3%). Unlike the findings of Defne et al. (12) in the Turkish population, tooth impaction was only observed in 1.25% of cases, making it the second least frequent factor. According to the literature, these cases of hypercementosis can be explained as a response to continuous tooth eruption (17 , 21). In our study, we also evaluated various local factors to a lesser extent. These factors included periodontal disease (4,03%), orthodontic treatment (3.87%), periapical infections (3,03%), faceting (2.52%), and endodontic issues (2.18%). Additionally, we noted the presence of other factors, such as deep caries, extensive restorations, dental malpositions, and maxillary lesions, which, combined, accounted for a total incidence of 8.4%. Regarding hypercementosis type, we observed that the diffuse form was the most frequent and the sleeve-shaped form was the least frequent, in agreement with other studies (5 , 12 , 20 , 22). The most affected teeth were the upper and lower molars, with a higher incidence in the maxilla (72%) than in the mandible (5 , 11 , 12 , 14 , 15), contrary to what is observed in the literature. Although the prevalence of hypercementosis in the lower incisors was low, two of the three cases were focal. Nevertheless, given the small sample size, we cannot conclude that lower incisors with hypoplastic enamel will always exhibit the focal type; this may be specific to the sample studied. However, this research did reveal a significant difference. In our research, although we can see significance between the association of local factors and the type of hypercementosis, with a higher incidence of focal or sleeve-shaped hypercementosis observed in cases where some factors coexist (endodontics, periapical lesion, increased bone density), there are few cases evaluated, so this association should be taken with caution. While our study found no significant association between the teeth affected by hypercementosis and sex, we observed that a higher proportion of the number of teeth with this condition occurred in women than in men. This observation was supported by multiple linear regression analysis, which suggested that being male might serve as a protective factor. We chose to use linear regression analysis to evaluate the number of teeth impacted by hypercementosis. In contrast, other studies often compare groups with and without hypercementosis, whereas our study included only cases that presented hypercementosis. Additionally, in most of the studies reviewed, sex was not a significant factor, except for one study conducted in a German population, which reported that females were 10 times more affected than males (14). As observed by Bernal Ruíz et al. (5) in the Mexican population and by Eren et al. (15) in the Turkish population, the majority of patients had only one tooth with hypercementosis (57.2%). Hypercementosis is a condition linked to a patient's age; however, in our study, age did not show a significant association (p=0.273). This finding aligns with the research conducted by Elsayed et al. (11) in Saudi Arabia, where age also had a non-significant correlation (p=0.633). Besides, our study found that the absence of an antagonist tooth was the local factor most commonly associated with hypercementosis. Interestingly, males demonstrated a lower incidence of hypercementosis, suggesting a possible protective effect regarding the number of affected teeth. Overall, age did not appear to be a significant factor, and diffuse hypercementosis was identified as the most prevalent type. One limitation of our study was the small sample size; we could have benefited from comparing it with a control group and from exploring additional interesting variables. While the results of this study cannot be generalized, the substantial sample size and methodology offer a solid foundation for formulating hypotheses for future research using different methodological approaches.

## Conclusions

The local factor most frequently associated with hypercementosis was the absence of an antagonist tooth. Males had a lower incidence of hypercementosis, which could be considered a protective factor for the number of teeth with this condition. Age was not a determining factor, and diffuse hypercementosis was the most prevalent type.

## Figures and Tables

**Table 1:** Number of teeth with hypercementosis in the sample evaluated in total and by sex.

Sex		Number of teeth with hypercementosis							Total
		1	2	3	4	5	6	7	
Female	n	138	54	13	10	3	2	0	220
	%	62.7	24.5	5.9	4.5	1.4	0.9	0.0	100.0
Male	n	61	41	12	9	3	1	1	128
	%	47.7	32.0	9.4	7.0	2.3	0.8	0.8	100.0
Total	n	199	95	25	19	6	3	1	348
	%	57.2	27.3	7.2	5.5	1.7	0.9	0.3	100.0

p=0.156 Chi-square test

**Table 2:** Type of teeth affected by hypercementosis in the total sample and by sex.

Sex		Affected tooth						
		Upper canines	Upper premolars	Upper molars	Lower incisors	Lower premolars	Lower molars	Total
Female	n	18	43	104	1	8	46	220
	%	8.2	19.5	47.3	0.5	3.6	20.9	100.0
Male	n	9	14	65	1	3	36	128
	%	7.0	10.9	50.8	0.8	2.3	28.1	100.0
Total	n	27	57	169	2	11	82	348
	%	7.8	16.4	48.6	0.6	3.2	23.6	100.0

p=0.272 Chi-square test

**Table 3:** Association between type of hypercementosis and sex.

Sex		Type of hypercementosis			
		Diffuse	Focal	Sleeve	Total
Female	n	202	13	5	220
	%	91.8	5.9	2.3	100.0
Male	n	117	8	3	128
	%	91.4	6.3	2.3	100.0
Total	n	319	21	8	348
	%	91.7	6.0	2.3	100.0

p=0.991

**Table 4:** Association between the type of hypercementosis and the tooth evaluated.

Type of tooth		Type of hypercementosis			
		Diffuse	Focal	Sleeve	Total
Upper incisor	n	1	0	0	1
	%	100.0	0.0	0.0	100.0
Upper canine	n	40	4	1	45
	%	88.9	8.9	2.2	100.0
Upper premolar	n	91	7	1	99
	%	91.9	7.1	1.0	100.0
Upper molar	n	273	8	2	283
	%	96.5	2.8	0.7	100.0
Lower incisor	n	1	2	0	3
	%	33.3	66.7	0.0	100.0
Lower canine	n	4	1	0	5
	%	80.0	20.0	0.0	100.0
Lower premolar	n	23	0	0	23
	%	100.0	0.0	0.0	100.0
Lower molar	n	122	8	6	136
	%	89.7	5.9	4.4	100.0
Total	n	555	30	10	595
	%	93.3	5.0	1.7	100.0

p<0.001 Chi-square test

**Table 5:** Associated factors in hypercementosis cases, and its association with type of hypercementosis.

Associated factor				n	%
Idiopathic				79	13.28
Impaction				125	21.01
Absence of antagonist				147	24.71
Faceting				15	2.52
Periodontal disease				24	4.03
Bone density				0	0.00
Periapical process				18	3.03
Endodontic treatment				13	2.18
Orthodontic treatment				23	3.87
Other factors				50	8.40
Combinations of factors				101	16.97
Total				595	100.00
Associated factor		Type of hypercementosis			
		Diffuse	Focal	Sleeve	Total
Idiopathic	n	77	2	0	79
	%	97,5	2,5	0,0	100
Impaction	n	123	2	0	125
	%	98,4	1,6	0,0	100
Absence of antagonist	n	146	1	0	147
	%	99,3	0,7	0,0	100
Faceting	n	12	3	0	15
	%	80,0	20,0	0,0	100
Periodontal disease	n	24	0	0	24
	%	100,0	0,0	0,0	100
Periapical process	n	13	4	1	18
	%	72,2	22,2	5,6	100
Endodontic treatment	n	12	1	0	13
	%	92,3	7,7	0,0	100
Orthodontic treatment	n	22	1	0	23
	%	95,7	4,3	0,0	100
Other factors	n	47	3	0	50
	%	94,0	6,0	0,0	100
Combinations of factors	n	79	13	9	101
	%	78,2	12,9	8,9	100
Total	n	555	30	10	595
	%	93,3	5,0	1,7	100

p<0.001 Chi-square test

**Table 6:** Association between the type of tooth with hypercementosis and the factors associated with it.

Associated factor		Type of tooth								
		Upper incisor	Upper canine	Upper premolar	Upper molars	Lower incisor	Lower canine	Lower premolars	Lower molars	Total
Idiopathic	n	0	13	27	12	1	2	7	17	79
	%	0,0	16,5	34,2	15,2	1,3	2,5	8,9	21,5	100
Impaction	n	0	7	2	59	0	0	1	56	125
	%	0,0	5,6	1,6	47,2	0,0	0,0	0,8	44,8	100
Absence of antagonist	n	0	0	13	119	0	0	6	9	147
	%	0,0	0,0	8,8	81,0	0,0	0,0	4,1	6,1	100
Faceting	n	0	8	3	0	2	1	1	0	15
	%	0,0	53,3	20,0	0,0	13,3	6,7	6,7	0,0	100
Periodontal disease	n	0	0	2	13	0	1	1	7	24
	%	0,0	0,0	8,3	54,2	0,0	4,2	4,2	29,2	100
Periapical process	n	0	2	2	10	0	0	0	4	18
	%	0,0	11,1	11,1	55,6	0,0	0,0	0,0	22,2	100
Endodontic treatment	n	0	1	4	4	0	0	4	0	13
	%	0,0	7,7	30,8	30,8	0,0	0,0	30,8	0,0	100
Orthodontic treatment	n	1	7	13	2	0	0	0	0	23
	%	4,3	30,4	56,5	8,7	0,0	0,0	0,0	0,0	100
Other factors	n	0	1	9	23	0	1	1	15	50
	%	0,0	2,0	18,0	46,0	0,0	2,0	2,0	30,0	100
Combinations of factors	n	0	6	24	41	0	0	2	28	101
	%	0,0	5,9	23,8	40,6	0,0	0,0	2,0	27,7	100
Total	n	1	45	99	283	3	5	23	136	595
	%	0,2	7,6	16,6	47,6	0,5	0,8	3,9	22,9	100

p<0.001 Chi-square test

**Table 7:** Multiple linear regression to evaluate the influence of sex and age on the number of teeth with hypercementosis.

Predictor variable	B	P	95.0% confidence interval for B	
			Lower limit	Upper limit
(Constant)	1.363	<0.001	.917	1.810
Sex	0.305	0.010*	.072	.537
Age	0.004	0.273	-,003	.012

*Significant

## Data Availability

The data supporting the findings of this study are available from the corresponding author upon reasonable request.
